# Trichobezoar in a young girl caused by ingestion of bristles brush for more than a decade: A case report

**DOI:** 10.1016/j.ijscr.2019.05.045

**Published:** 2019-07-11

**Authors:** Ahmad Al-Mouakeh, Mohammad Nour Shashaa, Muhamad Zakaria Brimo Alsaman, Aya Zazo, Mohamad shadi Alkarrash, Rama Zazo, Ammar Niazi

**Affiliations:** a Faculty of Medicine, University of Aleppo, Aleppo, Syria; b Surgery Department, Faculty of Medicine, Aleppo University Hospital, University of Aleppo, Aleppo, Syria

**Keywords:** Trichobezoar, Bowel obstruction, Hair, Bristle brush, Mass, Psychiatric disorders

## Abstract

**Introduction:**

Trichobezoar is a rare cause of bowel obstruction. In general, Trichobezoars are composed of hair and usually found incidentally in patients undergoing upper gastrointestinal endoscopy or imaging. Patients diagnosed with Trichobezoar may have psychiatric disorders.

**Discussion:**

Trichobezoars cause nonspecific symptoms like asymptomatic abdominal mass, vomiting, nausea, and anorexia. Diagnosis of Trichobezoar is made by endoscopic examination and radiological methods. Therapeutic options for trichobeazoar are chemical dissolution, endoscopic removal or surgery.

**Case presentation:**

Here we present a case of an 18-year-old girl who has a unique type of Trichobezoars caused by ingestion of hair and bristle clothes brush for 14 years. She presented to the surgical clinic complaining of vomiting, anorexia and epigastric mass. Abdominal computed tomography scanner showed nonattached intragastric mass which was consistent with trichobezoar. The patient was managed by surgical removal of the intragastric mass.

**Conclusion:**

Trichobezoar is caused by chronic ingestion of hair; it is commonly seen in young females who may have psychological disorders, such as trichophagia and trichotillomania. Common symptoms are abdominal pain, nausea, vomiting, and weight loss. Surgical intervention is performed for the majority of the patients.

## Highlights

• Ingestion of hair and bristle brush led to unique type of trichobezoars.
• Trichobezoars led to gastric outlet obstruction.
• The diagnosis is made by abdominal X-ray, computed tomography scan and endoscopic examination.
• Surgical intervention is performed for the majority of patients.

## 1 Introduction

Trichobezoar is a rare cause of bowel obstruction [1]. In history, bezoars from animal guts were used as precious stones and antidotes to poisons [2]. Bezoars can obstruct the digestive system, specifically the outlet of the stomach [3]. Bezoars are sorted according to their components: A bezoar comprised of milk products is identified as a lactobezor. A bezoar comprised of hair is identified as a trichobezoar. A bezoar comprised of vegetable materials is identified as a phytobezoar. A bezoar comprised of hair and food is identified as a trichophytobezoar. A bezoar comprised of cotton fibers is identified as a cotton bezoar. A bezoar comprised of medication is identified as a pharmacobezoar [4,5]. Trichobezoar is common in teenager females that usually have psychiatric disorders. If appropriate management was not applied, it may have bad prognosis with a high risk of death. 30% of mortality causes are due to gastrointestinal bleeding or perforation [6]. Here we present a case of Trichobezoar in an 18-year-old girl who used to ingest hair and bristle clothes brush for 14 years, and the main complaints were vomiting and anorexia.This work is reported accord to the SCARE criteria [7].

## 2 Case presentation

**Fig. 1:**
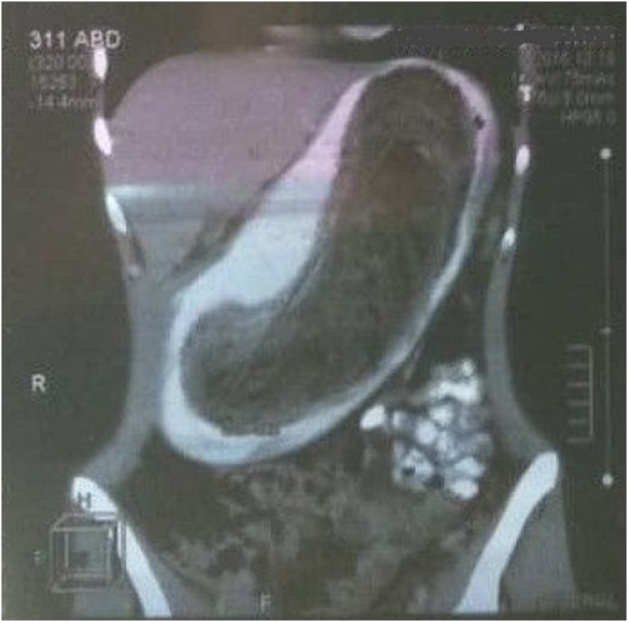
CT scan showing obstructer mass in the stomach.

**Fig. 2:**
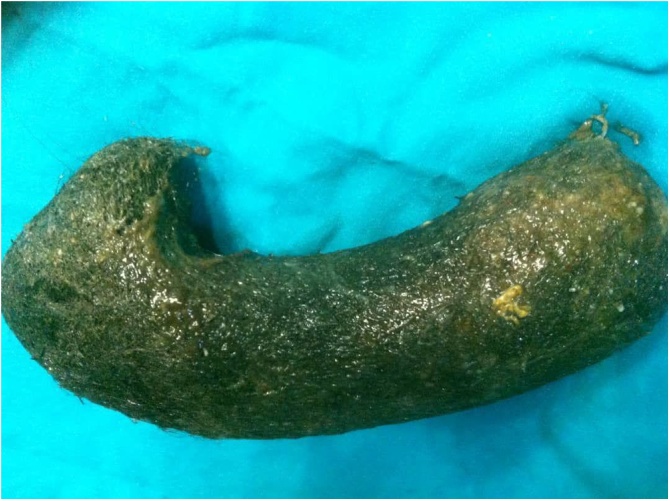
Large hairball 30 × 10 cm similar to stomach shape.

An 18-year-old girl with a body mass index (BMI) of 12.9Kg/m^2^ weight, presented to the surgical clinic with a one-month history of recurrent vomiting and anorexia. On observation, the patient was pale, fatigued and thin built. For the last 3 years, her nutrition was limited to fluids only. Abdominal examination revealed a painless, mobile mass extending from the epigastric to the umbilical region and it was visible in rest. Abdominal palpation showed no muscular defense. Laboratory studies revealed: Anemia (hemoglobin was 9 g/dl) and electrolyte abnormalities (hypocalcemia, hypokalemia). The patient had a history of anemia without any other gastrointestinal disease. The girl did not declare anything about hair ingestion, but her parents mentioned that she had a history of chewing hair. A CT scan with oral contrast was performed which showed a large non-attached intragastric mass (Fig. 1). Based on the patient's history of hair ingestion, physical examination, and CT scan, a diagnosis of trichobezoar was made and the patient underwent surgical removal of the intragastric mass. During surgery, excoriation and bleeding were observed in the mucus of the stomach. Anterior gastrotomy was performed, and large hairball mass (30 × 10 cm) that was occupying the whole stomach was removed (Fig. 2). After 6 months of follow up, the patient is doing well, her weight improved from 35 kg to 65 kg with a BMI of 23.8 Kg/m^2^, and now she is studying at college.

## 3 Discussion

Bezoar is a rare cause of gastric outlet obstruction. It is as a mass of foreign material within the gastrointestinal tract. Bezoars include many types: trichobezoar [hair fibers], phytobezoar [plant material], lactobezoar [milk curds] and miscellaneous [medications, tissue papers, shellac, tar, sand] [8]. We present a case of trichobezoar caused by ingestion of hair and bristle clothes brush which is a rare case in the medical literature. The vast majority of patients with trichobezoars have psychiatric disorders including trichotillomania and trichophagia. Other psychiatric disorders such as mental disorders, abuse, pica, obsessive-compulsive disorder, depression, and anorexia nervosa can be found [3]. The most common features of trichobezoar are abdominal pain, nausea, vomiting, early satiety, and weight loss [3,9,11]. Anemia, halitosis and alopecia have also been described [10,11]. On physical examination: well defined abdominal, smooth, firm and mobile mass in the epigastric region is an important suspecting finding [12]. Trichobezoar can cause many complications including gastric outlet obstruction, obstructive jaundice, gastrointestinal bleeding, protein-losing enteropathy, acute pancreatitis, small intestine obstruction, perforation, peritonitis, and intussusception [13,14]. When trichobezoar extends to small intestine it is called Rapunzel syndrome [10]. Trichobezoar diagnosis is made by endoscopic examination and radiological methods. Endoscopic examination plays a major role in diagnosis and treatment. In upper gastrointestinal endoscopy, trichobezoar appears as a colorful mass in the funds or antrum of the stomach. The examiner must be aware of the presence of gastric ulcers, perforation, or any other gastric pathology [15]. Radiological methods such as abdominal X-ray and computed tomography scan or ultrasonography show calcified, granular, or swirl like structures of solid and gaseous material or filling defects within the stomach. Diagnosis was confirmed by CT scan with oral contrast, which showed free-floating filling defects. Trichobezoar may suspect with malignancy, so it is important to exclude neoplasms as well [[16], [17], [18]]. Management of trichobezoar includes chemical dissolution, endoscopic removal, adjuvant prokinetics, and surgery. Chemical dissolution like Cola and adjuvant prokinetics like Metoclopramide can accelerate resolving of a gastric bezoar [19,20]. Endoscopic removal aims to break bezoars into small parts with the alternating use of a polypectomy snare and argon plasma coagulation [15]. In our case, surgical management was performed. In general, surgical therapy is considered when other ways cannot be performed or if there is a serious complication. Eventually, a review showed that laparotomy is the treatment of choice for trichobezoar but has a higher risk of complications [3].

## 4 Conclusion

Trichobezoar is caused by chronic ingestion of hair; it is commonly seen in young females who may have psychological disorders, such as trichophagia and trichotillomania. Common symptoms are abdominal pain, nausea, vomiting, and weight loss. Surgical intervention is performed for the majority of the patients.

## Conflicts of interest

The authors declare that they have no conflict of interest.

## Sources of funding

There are no sources of funding.

## Ethical approval

Not required for case reports at our hospital. Single case reports are exempt from ethical approval in our institution.

## Consent

Written informed consent was obtained from the patient for publication of this case report and accompanying images. A copy of the written consent is available for review by the Editor-in-Chief of this journal on request.

## Author’s contribution

Ahmad Al-Mouakeh: design of the study, data interpretation and analysis, revision.

Mohammad Nour Shashaa: data collection, revision, corresponding author.

Muhamad Zakaria Brimo Alsaman: design of the study, revising critically, wrote the manuscript.

Aya Zazo: patient care, data analysis, wrote the manuscript.

Mohamad shadi Alkarrash: data collection, data interpretation and analysis.

Rama Zazo: design of the study, patient care, revision.

Ammar Niazi: managed the patient and did the surgery, the Supervisor, patient care, revising critically.

All authors read and approved the final manuscript.

## Registration of research studies

N/A.

## Guarantor

Dr. Ammar Niazi.

## Provenance and peer review

Not commissioned, externally peer-reviewed.
